# Political Transition, Health System Reform and Trust in Childhood Vaccination in Ukraine: A Qualitative Case Study

**DOI:** 10.1002/hpm.70128

**Published:** 2026-08-25

**Authors:** Harriet Dwyer, Jennifer Palmer, Luisa Enria, Anton Grushetskyi, Olya Fokaf, Kateryna Gatsenko, Hanna Yahorava, Nadine Beckmann

**Affiliations:** ^1^ Department of Global Health and Development London School of Hygiene and Tropical Medicine London UK; ^2^ Kyiv International Institute of Sociology Kyiv Ukraine; ^3^ Independent Researcher

**Keywords:** childhood vaccines, conflict, corruption, health reform, Russia, soviet‐era governance, Ukraine

## Abstract

Trust in childhood vaccination is a concern for health policy, particularly in settings of political transition and conflict, where governance, institutional legitimacy and service delivery are often under strain. It is against this backdrop that this study aims to describe evolving interactions between health system governance, trust and childhood vaccination in Ukraine across periods of reform and conflict, and the implications for health policy in fragile settings. Drawing on qualitative interviews with key informants and Ukrainian parents, findings show that trust in the health system, with implications for vaccine confidence, has been intertwined with broader perceptions of the legitimacy of state institutions and quality of services. Trust was undermined by corruption and weak governance but reconfigured through reforms that increased accountability and patient choice. The 2022 full‐scale invasion disrupted services but also strengthened forms of civic responsibility and engagement with public health. This study contributes to broader scholarship on health system trust and vaccine confidence by situating them within the historical and political evolution of Ukraine. It offers considerations relevant for Ukraine's post‐conflict recovery and health governance and illustrates links between vaccine confidence and state‐society relations in contexts of political transition and crisis.

## Highlights

- Trust in vaccines in Ukraine is linked to perceptions of health system integrity and governance.
- Rapid political transition and persistent immunisation gaps in Ukraine provide an important context for understanding trust in vaccination.
- The full‐scale invasion has strengthened vaccine infrastructure and changed how participants described engagement with vaccination.
- Political and institutional context helps explain why trust in vaccines is still fragile today.

## 1 Introduction

Trust in vaccination is a concern for health policy in many settings [1]. The World Health Organization (WHO) has recognised vaccine hesitancy as a significant global public health challenge [2]. While recent developments in the United States have shown how trust in vaccination can be eroded through political contestation over public health and the governance of vaccine policy [3], contexts of political transition and conflict bring these dynamics into sharper focus by concentrating institutional change and uncertainty within health services.

Ukraine provides a clear example of such a context. From its inheritance of a highly centralised Soviet health system, which prioritised preventative public health and mass vaccination, to the country's renewed independence in 1991 and, since 2014, armed‐conflict with Russia, Ukraine has navigated periods of underfunded public health, systematic health reforms and societal transformation [4, 5].

Several key political moments have shaped community interactions with the Ukrainian health system. The 2004 Orange Revolution challenged authoritarian control over the media and reshaped Ukraine's geopolitical orientation [6], while the 2013/2014 Revolution of Dignity reignited aspirations for democratic change and European integration [7]. A major health reform process followed which saw the creation of the National Health Service of Ukraine (NHSU) and a decentralised, patient‐centred primary health care system [4]. More recently, Russia's full‐scale invasion of Ukraine in February 2022 has further strained the health system; exacerbating inequities while also reshaping new forms of social cohesion and civic engagement [8, 9].

Before the war, vaccination rates in Ukraine were among the lowest in Europe and since the full scale invasion there have been outbreaks of vaccine‐preventable disease including polio and measles [10]. There are an estimated 15,000 zero‐dose children (children who have not received any routine vaccination) in Ukraine [11]. The 2023 Ukraine Health Index revealed that 15.5% of respondents with children under 18 years reported refusing at least some vaccinations for their child[ren] [12]. While these indicators are often described in terms of vaccine hesitancy, there remains limited empirical research examining trust in vaccines within broader dynamics of health system governance. Whilst there have been some improvements in vaccination coverage across the country, persistent immunisation gaps remain [10, 11], raising questions about potential gaps in confidence in vaccines amongst other wider challenges of access and supply [1].

It is against this backdrop that this study aims to describe evolving interactions between health system governance, trust and childhood vaccination in Ukraine across periods of reform and conflict, and the implications for health policy in fragile settings.

## 2 The Health System in Ukraine: A Brief History

### 2.1 Soviet Era and Post‐Soviet Transition (Pre‐2014)

The USSR (Union of Soviet Socialist Republics) was a highly centralised, single party state that maintained strict control over political, economic and social life [13]. The Soviet public health system operated on principles that prioritised the public good and state control over individual preferences [5, 14]. Vaccination campaigns were a key component of this approach, fitting well with the ideology that valued the collective and sought to secure the health of the labour force [14]. While coercion was sometimes employed for public health measures, more common strategies included surveillance and social marketing, including public health campaigns aimed at prevention [14]. In Ukraine, the formal organisation structure of the health system aligned with the Soviet Semasko model. This model was characterised by central budgetary financing, hierarchical structure and the dominance of the public health sector [4, 5].

Following independence in 1991, Ukraine retained the core structure of the Soviet health system which faced chronic underfunding, institutional degradation and weak accountability [4, 15, 16]. Informal payments to health providers played an increasingly important role in health financing; helping health workers sustain their livelihoods in place of adequate salaries while causing burden to patient's household budgets [4, 17]. Attempts at significant financial and organisational reforms often failed due to a lack of strong political will, and the system appeared resistant to major policy reform [4, 18]. These persistent structural constraints formed part of wider conditions that contributed to public frustration and demands for political change expressed during the 2004 Orange Revolution [6].

Vaccine anxieties intensified within this context, reaching a peak during the WHO‐recommended 2008 measles‐rubella (MR) vaccination campaign [19]. The campaign, intended to vaccinate young people, was prematurely halted due to a highly publicised death erroneously linked to the vaccination, triggering political intervention and the suspension of the campaign [14, 19, 20]. Poor procurement decisions further undermined trust, including allegations involving the Minister of Health and her son's pharmaceutical company aiming to profit from vaccine tenders [21]. The episode became emblematic of how weak governance, politicised responses and poor communication could erode confidence in vaccination and the health system more broadly [14, 19, 20, 21].

### 2.2 Reform, Accountability and Changing State‐Citizen Relations (2014–2022)

The 2013‐2014 Revolution of Dignity represented a major political and institutional turning point [7, 22]. It signalled a rejection of the previous political course and served as a catalyst for democratic progress [15]. Subsequent reform efforts aimed to address entrenched corruption and rebuild public trust across state institutions including the health sector [23].

In 2015, to increase the availability of vaccines and eliminate abuses related to purchasing, Ukraine handed procurement of vaccines to international agencies including UNICEF, the United Nations Development Programme (UNDP), and UK contractor, Crown Agents [4, 24]. While a new National Strategy on Health aimed to improve the quality of, and access to healthcare and reduce corruption [4, 25]. Dr. Ulana Suprun, a US born radiologist with Ukrainian parents, was appointed as acting Minister for Health in 2016 and became the face of the reform, pushing for transparent, patient oriented care [26, 27].

A central element of the health reform was the establishment of the NHSU [23]. The NHSU became responsible for contracting health service providers and implementing a revised financing mechanism known as ‘money follows the patient’, which shifted funding away from Soviet‐style input based allocations toward output‐based reimbursement for services delivered [4, 23, 28]. This change was intended to improve efficiency and value for money within the health system [29].

At the primary healthcare level, reforms sought to reconfigure relationships between patients and providers [4, 23]. Patients were no longer assigned a doctor based on place of residence but were able to choose their primary care physician and formalise this relationship by signing a ‘declaration’ with provider reimbursement calculated according to the number of registered patients [4, 25, 30]. This process required registration through the national e‐health system, introduced in 2018, which enabled verification, reduced opportunities for duplicate enrolment and supported resource management [30]. Patients were able to change providers if dissatisfied, which acted both as a quality assurance tool and a deterrent against informal payments [23]. By early 2020, these reforms had shown encouraging indications of change, such as improved overall value for money, a reduction in out of pocket expenditures in primary care, and increased patient satisfaction with providers [29].

### 2.3 War With Russia (2014–2022)

Alongside domestic reform efforts, Ukraine's post 2014 trajectory unfolded under growing geopolitical tension following the annexation of Crimea by Russia and the onset of armed conflict in Eastern Ukraine [15, 31, 32]. During this period, Russian disinformation campaigns were used to confuse and undermine the information environment [33, 34, 35]. Since then, Russian disinformation in Ukraine, has consisted of a package of pro‐Russian ideas about geopolitics, domestic politics, and vaccines [36]. In the vaccination space these narratives have included both anti‐vaccination conspiracy theories, such as claims Western vaccines are unsafe or part of population control efforts, and pro‐vaccination messages linked to Russian made products like the Sputnik V COVID‐19 vaccine [36]. This dual strategy, often spread by trolls and bots, reflects a broader strategy of sowing confusion and political discord rather than promoting a coherent public health message [37, 38].

### 2.4 The Full‐Scale Invasion and System Shock (2022 Onwards)

Russia's full‐scale invasion of Ukraine in February 2022 placed unprecedented strain on the health system, disrupting routine services, displacing populations, and interrupting vaccination delivery and surveillance [16, 39, 40]. At the same time, substantial international support helped sustain core services through emergency financing, decentralised cold‐chain infrastructure and mobile vaccination teams [41, 42].

The full‐scale invasion has also contributed to shifting political and civic dynamics. Since February 2022, there has been a marked increase in national identification, civic engagement and trust in institutions [43]. Surveys conducted between 2022 and 2024 showed that an average of 80% of respondents identified themselves primarily as citizens of Ukraine, signalling a growing sense of unity across regional, linguistic and ethnic lines [43]. At the same time, the war has reshaped the media landscape, with over 70%–80% of Ukrainians now relying on digital platforms like Telegram and YouTube for information, compared to the pre‐2022 dominance of television; concurrently trust in traditional media has dropped to just 27% [44, 45]. Together, these shifts have fostered new forms of civic engagement and responsibility that may influence how citizens interact with public health institutions [9] and measures, including vaccination under wartime conditions, potentially shifting ways in which citizens interact with public health measures.

## 3 Methods

### 3.1 Study Design

This study used a qualitative design to examine the interactions between evolving health system governance, trust and childhood vaccination. Qualitative methods allowed us to generate contextualised insight into how these factors were understood and experienced by different actors within and around the health system. The semi‐structured interview method was used to enable participants to describe their experiences in their own words, while also allowing consistent exploration of the key dynamics of interest across all interviews.

In this study, we defined trust as a willingness to rely on another person or institution, accepting potential risk based on expectations of competence and good intentions [46]. We framed trust as an ongoing process that is shaped by social proximity, past experiences and broader institutional legitimacy [47, 48, 49]. It is a form of social cohesion, rooted in shared meaning, and collective orientation, hence it can be fragile, difficult to build and easily fractured in settings of political or social disruption [50].

Vaccine confidence was treated as a specific expression of trust, referring to confidence in vaccines themselves as well as in the actors and institutions responsible for their development, delivery and recommendation [51]. These concepts informed the design of the interview guides and interpretation of participants accounts.

Data were collected through semi‐structured interviews with 21 key informants (journalists, academics, government officials, humanitarian responders and civil society) and 30 Ukrainian parents residing in Ukraine and Poland.

### 3.2 Study Setting

Ukraine was selected as the study setting because it represents a case in which political transition, health system reform and armed conflict have unfolded over a relatively short period, allowing examination of different governance conditions alongside dynamics of trust and childhood vaccine confidence.

The study was conducted in Ukraine and Poland to capture both in‐country perspectives and the experiences of displaced Ukrainian parents following the large‐scale population movements triggered by Russia's full‐scale invasion [52] and the subsequent humanitarian public health response.

### 3.3 Participant Recruitment and Consent

Purposive sampling was used to recruit participants with relevant experience across the study groups, with snowball sampling subsequently used to identify additional participants through existing networks. Three groups of parents were identified; *(i) parents displaced to Lviv from other parts of Ukraine (ii) those already residing in Lviv prior to the full‐scale invasion and (iii) parents who had left Ukraine and temporarily settled in Warsaw since the full‐scale invasion* (detailed in Table S1). These groups were selected to capture a range of experiences from different parts of Ukraine, including those having varied experiences amid the ongoing war. Initial recruitment of families displaced to Lviv, was done through temporary accommodation shelters across the city. Access was facilitated through non‐governmental organisations (NGOs) providing services to displaced families. Parents residing in Lviv over the longer term, were recruited in community spaces around the temporary facilities including churches, activity centres for children and online through Facebook community groups for local parents. In Warsaw, parents were reached through services for displaced Ukrainians to Poland including child friendly spaces and a Ukrainian kindergarten. Initial participants recommended other participants.

Key informants were purposefully sampled to generate a range of perspectives including representatives from the Ukrainian government (national and local), humanitarian organisations, the media, academia and the health sector. This approach aimed to facilitate exploration of trust across different levels of the health system, including policy, service delivery, public communication and humanitarian response.

Recruitment aimed to generate a sufficiently rich and diverse dataset to address the study aim while maintaining a manageable volume of data for detailed thematic analysis. The objective was not to produce a statistically representative sample, but to develop contextualised insights across different experiences of health system engagement. Informed consent was obtained from all participants. Given the potential for mistrust of outsiders and authorities, the process was guided by local members of the research team.

### 3.4 Data Sources

For interviews, topic guides were informed by the related literature and consultations with public health practitioners currently working in Ukraine. Topic guides were iterative, evolving with the collection of data covering (i) political and crisis context (ii) access to vaccines and service delivery (iii) decision making around childhood vaccination (iv) trust in institutions and actors and (v) information flows and community dynamics.

Data were collected between November 2024 and March 2025 including several weeks of fieldwork in Ukraine (November‐December 2024) and a subsequent period of data collection in Poland (February‐March 2025). Semi‐structured interviews were conducted in person by the lead author and two research assistants who also acted as interpreters. Key‐informant interviews were conducted either in person or via zoom. Following each interview, the lead researcher and research assistants undertook a structured debrief, with reflections documented in a fieldwork diary. All interviews were audio recorded and transcribed in the language in which they were conducted and then translated into English by a professional translator. Any uncertainties in translation or interpretation were discussed during researcher debriefs or the transcription process to preserve the contextual meaning for analysis.

### 3.5 Analysis

Data analysis began during fieldwork and was guided by an iterative and reflexive process. After each interview, HD debriefed with KG in Ukraine and HY in Poland, emerging insights were compared and reflections noted in a fieldwork diary. These reflections shaped both the evolving topic guide and the preliminary analytical direction.

A six phase thematic analysis was undertaken to explore themes across the dataset [53]. This involved initial hand coding of the data during immersion then line by line coding of electronic transcripts and field notes in NVivo v.12 [54]. Coding was undertaken by HD and discussed regularly with the wider research team throughout the analytical process. Initial codes were generated inductively from participants accounts. Initial coding provided a structure for the analysis through codes in the areas of (i) institutional trust, (ii) health system navigation, (iii) wartime disruption and (iv) vaccine decision‐making. They were iteratively reviewed and refined through repeated engagement with the data set to develop coherent themes. This process involved comparison across interviews, field notes and researcher reflections, allowing for patterns to be explored across participants and study settings. Candidate themes were reviewed and discussed within the research team to ensure they were grounded in the dataset before being refined into the final analytical themes during the writing process. Data from the two country contexts were analysed both separately and together, allowing for triangulation of key themes. As part of the analytical process, a timeline (see Figure 1) was created to map key events and public health moments against narratives presented in interviews.

**FIGURE 1:**
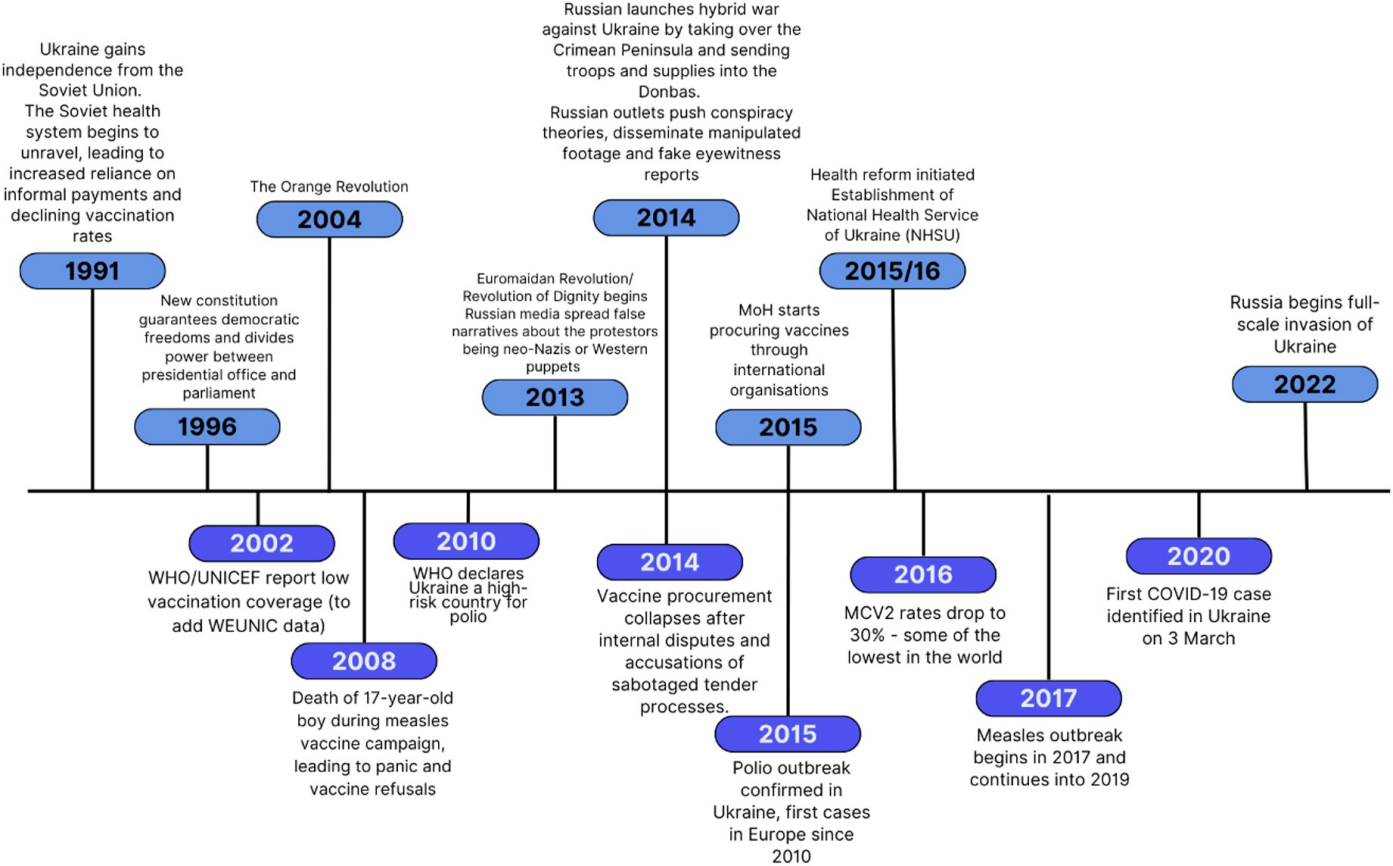
Timeline of key political (above light blue), and public health events (below dark blue) in Ukraine 1991–2022. (MCV2 refers to measles‐containing‐vaccine second‐dose) [6, 7, 20, 24, 31, 36, 55, 56].

### 3.6 Ethics

Ethics approval was obtained from the London School of Hygiene & Tropical Medicine Observational Research Ethics Committee (ref: 29965), The Institutional Review Board #1 of the Ukrainian Institute of Public Health Policy (ID: 2024‐015‐01), and the Bioethics Committee of the Institute of Mother and Child in Poland (Resolution: No. 62/2024).

### 3.7 Reflexivity Statement

This research was conducted by a team combining both insider and outsider perspectives [57, 58]. Building a collaborative, multinational authorship team was a deliberate part of the study design. Researchers from LSHTM were not from Ukraine or the region, and therefore relied on the expertise of researchers from Ukraine and Belarus to contextually ground the study through their lived experience, linguistic fluency and deep local knowledge. Interviews were conducted by the lead author, together with local members of the research team, whose familiarity with the language and context supported participant engagement and interpretation. Throughout data collection and analysis, the research team held debriefs to reflect on emerging findings, positionality and potential influences on interpretation through individual assumptions. We acknowledge that these varied positionalities shaped the research process, influencing both participant engagement and data interpretation.

## 4 Findings

The historical and political developments outlined above (see Figure 1) provide the backdrop against which key informants and parents described their experiences with Ukraine's health system and their perceptions of vaccination in interviews. Descriptive characteristics of participants are detailed in Table S1.

Drawing on interviews, this section presents four empirical themes reflecting how participants described trust, vaccination and engagement with the health system. These themes capture recurring patterns in participants' accounts and are later interpreted in the Discussion in relation to the broader political history. They are (*i) corruption as a driver of mistrust; (ii) democratic transition, reform and the reconfiguration of trust (iii) agency, choice and changing relations with the health system, and (iv) war with Russia and the changing nature of vaccine confidence*.

### 4.1 Corruption as a Driver of Mistrust

Across interviews, corruption emerged as a persistent feature shaping public engagement with Ukraine's health system. Key informants and parents described informal payments, low salaries and opaque procurement practices as long‐standing characteristics of healthcare delivery, contributing to low institutional trust. As one key informant explained:

Ukraine always had very low institutional trust, because before, it was Soviet Union … then it was after independence, institutions that were very imperfect and very problematic. In general, people were separated from authorities, and all authorities were perceived as the corrupt guys.

[KII12]

Several key informants linked everyday corruption within healthcare to systemic underfunding and low wages, which in turn normalised informal payments:

There were billions and billions of *hryvnia* [Ukrainian currency] being taken, stolen from the system itself… physicians had these very low salaries, and they were taking cash under the table, undeclared.

[KII5]

In the vaccination context, corruption was most frequently described in relation to procurement and certification practices. Key informants described vaccine procurement prior to reform as involving ‘collusion everywhere along the way’ [KII5], with pharmaceutical companies reportedly influencing tender processes. These practices were associated with concerns about vaccine quality, including accounts of low quality or improperly handled vaccines entering the system. Parents similarly linked procurement practices to their own considerations about vaccination. One parent described the Ministry of Health during the 2008 measles‐rubella campaign as ‘an absolutely corrupt agency’, concluding:

Here we could not put the health of our children at great risk.

[Parent 13]

Across these accounts, concerns about corruption were not expressed as abstract critiques of governance but concrete experiences and observations including falsified vaccination certificates, informal payments and different levels of trust in state‐provided versus privately purchased vaccines. In this context, mistrust was directed less at vaccination as a medical intervention and more at the institutions responsible for its procurement, oversight and delivery.

### 4.2 Democratic Transition, Reform and the Reconfiguration of Trust

Key informants described Ukraine's early post‐independence years as marked by political instability and governance challenges, with implications for how childhood vaccination was understood. As the country moved away from the Soviet system in the early post‐independent period, vaccine hesitancy was described in interviews as having political rather than medical origins, most visibly during the 2008 MR vaccine incident:

All this hesitation, especially, in the early Ukrainian independence period when … this process has political tension, and political manipulation.

[KII 11]

Interviewees consistently identified the 2013‐2014 Revolution of Dignity, and subsequent reform as a turning point. Trust in the state and in vaccines was seen as part of a broader transformation towards democratic accountability:

I would say that the changes here began, in fact, since the Revolution of Dignity, when the medical system began to be reformed … when you start to trust your society more, it cannot *but* affect this sphere.

[Parent 13]

Many interviewees named Dr. Suprun as a key figure leading this transformation. She was often contrasted favourably with other former Ministers of Health and described as both an outsider with Western expertise and connected with the Ukrainian diaspora. The dual positioning lent her credibility.

When Ms. Suprun came to us and saw the level of our medicine, when it was just iron beds, shabby walls, lack of medicines, she simply said: this should not be the case.

[KII18]

Participants associated her tenure with visible reform efforts, increased communication and greater international engagement:

…colossal changes began. And a lot of work was done, including replacing doctors in the field, and trust in what was happening actually increased.

[Parent 13]

… there was a huge information campaign on vaccination, international donors were involved.

[KII3]

At the same time, participants emphasised that changes in trust were gradual and uneven, unfolding over multiple political cycles rather than a single rupture:

It is a bigger problem of building trusting relations… some of these problems began to be corrected after the Orange Revolution of 2004, some after 2014, some are even being corrected now. But this path will take a very long time.

[KII4]

### 4.3 Agency, Choice and Changing Relations With the Health System

Several participants reflected on how historical experiences, particularly during the Soviet Union, shaped enduring patterns of disengagement from the medical system. Medicine was described as coercive, with long term effects on how people related to healthcare institutions, including avoiding contact where possible as describe by one parent:

In the Soviet Union… people tried not to come into contact with it [the medical system] at all…. Over these 30–40–50 years, a certain cultural layer has been created … you continue to avoid relations with the medical system.

[Parent 13]

Against this backdrop, the post‐2016 health reform was described by many participants as a critical moment for shifting norms, by introducing choice and agency into healthcare interaction:

In my opinion… the reform has given people a choice.

[KII16]

One of the most notable changes identified by interviewees was the empowerment of primary care providers, particularly those operating independent practices which was seen to improve accountability and patient trust:

The reform allowed primary care doctors to conduct their own practices, and doctors who have their own practice have a completely different attitude towards vaccination than doctors in state and municipal institutions. That is, they have greater responsibility.

[KII13]

Across accounts, the shift from centralised assignment towards patient choice and provider autonomy was described as changing expectations on both sides of the clinical encounter. Participants linked these changes with greater engagement with healthcare services and, for some, increased confidence in vaccination.

### 4.4 War With Russia and the Changing Nature of Vaccine Confidence

Participants described the full‐scale invasion as disrupting some aspects of vaccine delivery while strengthening others, with mixed implications for vaccine confidence. In the early stages of the invasion, immediate survival needs were described as taking precedence over preventative health, as described by a key informant:

Such challenges [such as a decrease in the number of vaccinations due to hostilities, lack of energy supply, etc.] really existed in 2022… the population's attention immediately shifted to much more immediate things than prevention.

[KII 8]

At the same time, several key informants described what they perceived as a broader shift in citizens' relationship with public institutions following the full‐scale invasion [KII4, KII8, KII10, KII12.] Rather than discussing vaccination solely as an individual health behaviour, these participants linked it to wider ideas of active citizenship, collective responsibility and Ukraine's aspirations towards European governance and public institutions. In these accounts, responsibility extended beyond protecting one's own child. It reflected participation in institutional systems now more associated with democratic governance and collective responsibility. One key informant articulated a tension between heightened collective orientation and the practical uncertainties of daily life under wartime conditions:

On the one hand, lots of people were influenced by the belief that we are finally part of the western civilization … and they should behave properly. In this case, for lots of people, it's saying, ‘We should reach some higher level. It also includes better healthcare that should also include regular checkups, vaccination…’ On the other hand, it's [the war] ruining everyday lives and lots of people postpone vaccination, not because they're against it but because of this uncertainty. You're living within one day; you're trying to survive only one day.

[KII 12]

Alongside these shifts in perception, key informants consistently pointed to significant wartime investments in immunisation infrastructure, particularly the resilience of the cold chain:

Now…we have completely updated the cold chain at the regional level… modern, powerful, qualified cold storage rooms … modern refrigerator trucks … Taking into account the constant shelling and destruction of the power system, we have ensured uninterrupted power supply.

[KII 8]

The war gave a chance, of course, to the huge upgrade of the cold chain … even when there are blackouts. I would say the big revolutionary change during the war is the cold chain equipment.

[KII 10]

Despite these improvements, parents and key informants described persistent uncertainty about vaccine safety under wartime conditions, particularly in relation to storage, transport and power outages:

Currently, the main reasons [for] distrust of vaccines [are] distrust of how they are transported and labelled … perhaps people have developed a fear of how these vaccines are stored in these conditions during the power outages.

[KII 3]

We also had the power outage, I worried about the quality of the vaccine, whether the pharmacy or clinic kept it in proper condition… not all pharmacies were immediately equipped with generators.

[Parent 15]

… those are just rumours. I don't know if they are true or not. Some people said that there was the whole fridge of spoilt vaccines… I don't know. Maybe true. Maybe not.

[Parent 18]

Several participants also pointed to the longer‐term impacts of the ongoing war with Russia, which since 2014, has included an information dimension. Participants described vaccine‐related disinformation as part of the broader information environment:

Of course, some [vaccine hesitancy] has its roots in us [public health actors], because we don't do our job perfectly… but also the roots of this misconception … is also another side of the war, because this is also the misinformation from our neighbours [Russia].

[KII 9]

Across these accounts the full‐scale invasion was described as simultaneously strengthening technical capacity while intensifying uncertainty in everyday decision making. Participants highlight how improvement in infrastructure did not always translate into confidence at the individual level, particularly in a context shaped by prolonged conflict and a contested information space.

## 5 Discussion

Our results show that different forms of trust within the health system in Ukraine has evolved alongside political transition, health system reform and conflict, with implications for vaccine confidence. This discussion situates the findings withing Ukraine's recent political and institutional history and considers their relevance for health policy.

### 5.1 Governance Legacies, Corruption and Institutional Mistrust

In Ukraine, low confidence in vaccines was articulated not as an individual or irrational trait, but as a historically situated trust response to institutional weaknesses and corruption. These dynamics echo what Ukrainian sociologists Golovakha and Panina [60] described in the early 2000s as ‘double institutionalisation’, in which formal state services operated in parallel with informal or private arrangements dependent on the same personnel. This blurring of public duty and personal gain undermined institutional legitimacy and fostered an oppositional ‘us‐versus‐them’ orientation, in which citizens positioned themselves against unaccountable authorities, a pattern explicitly articulated by key informants in this study.

These patterns are not unique to Ukraine. They align with wider scholarship that conceptualises corruption in health systems as a structural phenomenon rather than a series of individual moral failures. As Hutchinson et al. [61] argue, informal practices often emerge as workarounds in underfunded and bureaucratically constrained systems; while they may serve short‐term functional needs, they ultimately erode the legitimacy of the system [62].

In Ukraine, the 2008 MR vaccine crisis became emblematic of this. Public anxieties around vaccine safety were entangled with broader political mistrust and deeper suspicions towards the health system, amplified by allegations of corrupt procurement practices and politicised responses by state authorities [14, 20]. Rather than being interpreted solely as biomedical failure, the episode reinforced perceptions of institutional unreliability. These findings suggest that confidence in vaccines may function as a proxy for trust in the health system, with vaccination decisions reflecting broader evaluations of institutional integrity.

### 5.2 Political Progress, the Health System and the Reconfiguration of Trust

While the legacies of corruption were linked to public mistrust in the health system, participants consistently identified the post‐2014 period as a turning point in how trust began to be renegotiated. Many pointed to the Revolution of Dignity as a critical inflection point, aligning with wider accounts of post‐2014 governance reforms in Ukraine [23, 30]. Reforms such as the establishment of the NHSU, the outsourcing of vaccine procurement, and the institutionalisation of patient choice were described as concrete signals of greater accountability in the health system. Dr. Suprun, who drove the reform, was frequently referenced by participants as embodying this shift towards transparency and system change [26, 27].

Accounts from parents and key informants illustrate how these reforms reconfigured the social meaning of engagement with the health system. The ability to choose one's own doctor and avoid informal payments, were not merely bureaucratic improvements but were experienced as a restoration of dignity and agency in encounters with public institutions. In this context, trust emerged as a relational process, produced through everyday interactions between patients, providers and the wider health system. Importantly, participants did not describe trust as a single, undifferentiated construct. Trust in family doctors and everyday interactions with the health system could coexist with scepticism towards political institutions or the broader state. This reinforces the importance of distinguishing between interpersonal and institutional trust, while recognising that these different forms of trust remain closely interconnected [63, 64]. The increased relational proximity associated with these reforms appeared to create conditions in which vaccine acceptance *could* reflect confidence in the broader integrity of the system. However, as participants accounts also suggest, this trust remained fragile and uneven, requiring ongoing institutional effort to sustain [64].

### 5.3 War, Uncertainty and Civic Action

The full‐scale invasion disrupted these gains while also influencing interactions with the health system. Participants reflected how the realities of war affected their prioritisation of health. As immediate concerns around safety, displacement and day‐to‐day survival became dominant, preventative healthcare including childhood vaccination, was sometimes deferred. This highlights that lower vaccination uptake in conflict settings may reflect competing priorities rather than declining confidence alone, highlighting the importance of distinguishing between practical constraints and vaccine confidence when interpreting immunisation trends in crisis contexts.

Alongside these changes in health seeking behaviour, the rapid scaling of cold chain infrastructure, often in partnership with international agencies, demonstrates the capacity of the wartime system to innovate under pressure [39]. However, as our findings suggest, these improvements in infrastructure did not always translate into strengthened confidence. Doubts about vaccine storage and safety reflect a gap between technical resilience and perceived competence. This echoes literature on crisis‐affected communities where information vacuums and historical mistrust can amplify uncertainty even in the presence of functioning systems [1].

Several key informants described what they perceived as changing relationships between citizens, the state and public health since the full‐scale invasion. Vaccination became associated with broader commitments to collective wellbeing and civic engagement with public institutions. Piotr Sztompka [65] echoes this in his work by articulating this as a ‘bright side’ of the tragedy of war, with the creation of new opportunities for collective action, solidarity and public engagement with institutions. Similar dynamics have been observed in other health emergencies, where engagement with vaccination has served as a way for citizens to articulate belonging, responsibility and moral commitment to the nation during moments of existential threat [66, 67]. This aligns with Goldenberg's [50] framing of childhood vaccination as a civic act, reflecting a willingness to engage with a collective endeavour grounded in legitimacy and mutual responsibility [68]. In our study, this theme emerged primarily from key informant accounts and should be understood as an emerging narrative rather than a universal experience among participants. Where present, these forms of civic engagement [9] may offer opportunities to sustain public trust.

Taken together, these findings reinforce arguments that trust in vaccines is not simply a matter of communication or access, but of governance and social contract [69]. In Ukraine, participants described trust as being negotiated against the backdrop of historical experiences of institutional failure and contemporary democratic aspirations. These findings suggest that rebuilding confidence requires more than biomedical assurance; it depends on whether health systems are experienced as legitimate, equitable and worthy of citizens' trust [64, 69].

### 5.4 Strengths and Limitations

This study's primary strength lies in situating vaccine confidence in Ukraine within its broader political, historical and institutional context. However, limitations should be acknowledged. First, the analysis draws on retrospective interpretation of political events and health system change. These narratives are inevitably shaped by present circumstances, particularly the full‐scale invasion, which may influence how earlier periods are recalled and interpreted. Second, while participants frequently linked health system reform and political change to shifts in vaccine confidence, these associations reflect participants' perceptions rather than causal relationships, which this study was not designed to establish.

Third, the concept of ‘trust’ itself operates at multiple levels; institutional, interpersonal, and systemic, and while this analysis embraced that complexity, participants may have conflated these dimensions in ways that complicate the interpretation. Fourth, most parent participants were women, reflecting caregiving roles and patterns of displacement during the war; fathers' perspectives are therefore underrepresented. Finally, although the study includes parents from different parts of Ukraine, including those displaced from active conflict zones in eastern Ukraine, it does not capture experiences across all parts of the country. Perspectives may differ in regions under prolonged occupation or more directly affected by ongoing active conflict. As such, the analysis should be read as illustrative rather than exhaustive. Despite these limitations, the study offers timely, grounded insights into how trust in vaccination evolves in settings of political transition, reform and conflict.

### 5.5 Considerations for Policy and Practice

The findings of this study highlight that trust in vaccines is deeply embedded in the historical relationships between individuals, the state and the health system. Policy efforts should, therefore, address both the quality of health service delivery and the contextual dimensions of trust. This includes:

- Valuing agency and free choice by enabling individuals and communities to exercise meaningful control over their healthcare decisions.
- Recognising the role of relational trust, particularly between healthcare providers and their patients, and supporting this relationship through adequate funding and policy for primary healthcare providers.
- Situating vaccine policy within broader governance reform, with an emphasis on equity, transparency and accountability, recognising that confidence in vaccines reflects judgements about institutional integrity.
- Avoiding the instrumentalisation of vaccination in political discourse, as continued politicisation undermines public trust.
- Leveraging positive narratives of vaccination as civic value, framing vaccination not only as a biomedical intervention but as a collective practice linked to social responsibility, mutual care and participation in public life.

## 6 Conclusion

Trust in vaccines in Ukraine cannot be disentangled from the country's political history, health system transformation and experiences of governance. This study shows that trust in the health system, with implications for vaccine confidence, has evolved through legacies of corruption, moments of reform and systemic shifts in relationships between citizens and public institutions caused by the full‐scale invasion. As Ukraine navigates recovery and future reform, the challenge will be not only to deliver vaccines, but to continue building a health system that citizens see as trustworthy, accountable and their own. Beyond Ukraine, this case illustrates how vaccine confidence is connected to political and historical foundations of trust in public institutions, and how moments of crisis can reconfigure citizen‐state relations with implications for public health. Together with a companion paper examining information ecosystems [70], these findings highlight the importance of understanding vaccination as embedded within broader political, institutional and social processes.

## Author Contributions

**Harriet Dwyer:** conceptualisation, formal analysis, investigation, project administration, writing – original draft, writing – review and editing. **Jennifer Palmer:** supervision, methodology, writing – review and editing. **Luisa Enria:** supervision, methodology, writing – review and editing. **Anton Grushetskyi:** writing – review and editing. **Olya Fokaf:** writing – review and editing. **Kateryna Gatsenko:** investigation, project administration. **Hanna Yahorava:** investigation, project administration. **Nadine Beckmann:** supervision, methodology, writing – review and editing, project administration. All authors have read and approve the final manuscript.

## Funding

Harriet Dwyer, a PhD candidate, received support from the London School of Hygiene and Tropical Medicine through a Travelling Scholarship, which contributed to the fieldwork for this study.

## Ethics Statement

Ethics approval was obtained from the London School of Hygiene & Tropical Medicine Observational Research Ethics Committee (ref: 29965). The Institutional Review Board #1 of the Ukrainian Institute of Public Health Policy (ID: 2024‐015‐01), and the Bioethics Committee of the Institute of Mother and Child in Poland (Resolution: No. 62/2024).

## Conflicts of Interest

The authors declare no conflicts of interest.

## Supporting information


**Table S1:** Participant characteristics.

## Data Availability

All relevant data for this study are publicly available from the UK Data Service ReShare repository (http://doi.org/10.5255/UKDA‐SN‐858529).
