# Supplementary material for: Political Transition, Health System Reform and Trust in Childhood Vaccination in Ukraine: A Qualitative Case Study
Source: Int J Health Plann Manage. 2026 Aug 25;41(6):1277–88. doi: 10.1002/hpm.70128 (PMC13730252; doi:10.1002/hpm.70128)
Supplement: Supplementary file 1 [file HPM-41-1277-s001.docx]

**Supplementary information**

**Table a: Participant characteristics**

| **Semi-structured interviews with parents** | | | | |
| --- | --- | --- | --- | --- |
| **ID** | **Location** | **Status** | **Sex** | **Date of interview** |
| Parent 1 | Lviv, Ukraine | IDP | F | 7/12/2024 |
| Parent 2 | Lviv, Ukraine | IDP | F | 7/12/2024 |
| Parent 3 | Lviv, Ukraine | IDP | F | 7/12/2024 |
| Parent 4 | Lviv, Ukraine | IDP | F | 7/12/2024 |
| Parent 5 | Lviv, Ukraine | IDP | F | 7/12/2024 |
| Parent 6 | Lviv, Ukraine | IDP | F | 7/12/2024 |
| Parent 7 | Lviv, Ukraine | IDP | F | 7/12/2024 |
| Parent 8 | Lviv, Ukraine | IDP | F | 8/12/2024 |
| Parent 9 | Lviv, Ukraine | IDP | F | 8/12/2024 |
| Parent 10 | Lviv, Ukraine | IDP | F | 9/12/2024 |
| Parent 11 | Lviv, Ukraine | IDP | F | 9/12/2024 |
| Parent 12 | Lviv, Ukraine | IDP | F | 10/12/2024 |
| Parent 13 | Lviv, Ukraine | Lviv resident prior to 2022 | F | 7/12/2024 |
| Parent 14 | Lviv, Ukraine | Lviv resident prior to 2022 | F | 8/12/2024 |
| Parent 15 | Lviv, Ukraine | Lviv resident prior to 2022 | F | 9/12/2024 |
| Parent 16 | Lviv, Ukraine | Lviv resident prior to 2022 | F | 9/12/2024 |
| Parent 17 | Lviv, Ukraine | Lviv resident prior to 2022 | M | 10/12/2024 |
| Parent 18 | Lviv, Ukraine | Lviv resident prior to 2022 | F | 10/12/2024 |
| Parent 19 | Lviv, Ukraine | Lviv resident prior to 2022 | M | 10/12/2024 |
| Parent 20 | Lviv, Ukraine | Lviv resident prior to 2022 | F | 10/12/2024 |
| Parent 21 | Warsaw, Poland | Displaced to Warsaw after 2022 | F |  |
| Parent 22 | Warsaw, Poland | Displaced to Warsaw after 2022 | F |  |
| Parent 23 | Warsaw, Poland | Displaced to Warsaw after 2022 | F |  |
| Parent 24 | Warsaw, Poland | Displaced to Warsaw after 2022 | F |  |
| Parent 25 | Warsaw, Poland | Displaced to Warsaw after 2022 | F |  |
| Parent 26 | Warsaw, Poland | Displaced to Warsaw after 2022 | F |  |
| Parent 27 | Warsaw, Poland | Displaced to Warsaw after 2022 | F |  |
| Parent 28 | Warsaw, Poland | Displaced to Warsaw after 2022 | F |  |
| Parent 29 | Warsaw, Poland | Displaced to Warsaw after 2022 | F |  |
| Parent 30 | Warsaw, Poland | Displaced to Warsaw after 2022 | F |  |
| **Key informant interviews** | | | | |
| **ID** | **Location** | **Sector** |  | **Date** |
| KII 1 | Kyiv | Public health |  | 30/11/2024 |
| KII 2 | Kyiv | Public health |  | 30/11/2024 |
| KII 3 | Kyiv | Media |  | 7/12/2024 |
| KII 4 | Lviv | Media |  | 11/12/2024 |
| KII 5 | Kyiv | Government and public health |  | 18/12/2024 |
| KII 6 | Kyiv | Government and public health |  | 12/12/2024 |
| KII 7 | Kyiv | Government and public health |  | 11/12/2024 |
| KII 8 | Lviv | Government and public health |  | 12/12/2024 |
| KII 9 | Kyiv | Humanitarian response |  | 15/01/2025 |
| KII 10 | Kyiv | Humanitarian response |  | 9/01/2025 |
| KII 11 | Lviv | Academia |  | 9/12/2024 |
| KII 12 | Kyiv | Academia |  | 10/12/2024 |
| KII 13 | Lviv | Health worker 1 |  | 8/12/2024 |
| KII 14 | Lviv | Health worker 2 |  | 10/12/2024 |
| KII 15 | Lviv | Health worker 3 |  | 10/12/2024 |
| KII 16 | Lviv | Health worker 4 |  | 10/12/2024 |
| KII 18 | Warsaw | Health worker 5 |  | 13/02/2025 |
| KII 19 | Warsaw | Health worker 6 |  | 13/02/2025 |
| KII 20 | Warsaw | Health worker 7 |  | 25/02/2025 |
| KII 21 | Warsaw | Humanitarian response |  | 6/02/2025 |
